# Crime generators or social capital organizations? Examining the effects of places of worship on neighborhood crime

**DOI:** 10.1371/journal.pone.0282196

**Published:** 2023-03-08

**Authors:** James C. Wo

**Affiliations:** Department of Sociology and Criminology, The University of Iowa, Iowa City, IA, United States of America; University of Massachusetts Lowell, UNITED STATES

## Abstract

Places of worship (POW) have traditionally been argued to have crime-reducing effects in neighborhoods because of their ability to produce social capital. Yet, the evidence for this proposition is surprisingly weak. Consequently, an alternative proposition, rooted in environmental criminology, suggests that POW might unintentionally operate as crime generators in neighborhoods insofar as they induce foot traffic and undermine guardianship and social control capabilities. Because of these competing propositions in combination with the limited number of studies on this topic, we conduct a block group analysis of crime, places of worship, well-established criminogenic facilities, and sociodemographic characteristics in Washington, DC. We estimate negative binomial regression models of both violent and property crime and find strong evidence for only one of the propositions, with the effects of POW being relatively strong in comparison to other predictors in the models. The implications of these findings for criminology, urban studies, and public policy are discussed.

## Introduction

Places of worship (POW) broadly refer to locations where people gather to practice some type of religion, such as Christianity, Hinduism, Islam, and Judaism, to name a few. In addition to religious socialization, research has shown that POW are important because they tend to facilitate social ties, mutual cohesion and trust, and a willingness to intervene for the common good [1–5], which In turn, can be used instrumentally to achieve a collective goal, namely minimizing crime in neighborhoods. The key implication is that social capital that originates within POW extends to other settings in the larger community [6–9], ultimately strengthening informal social control mechanisms, such as the dissemination of information, mobilization of resources, or an informal system of monitoring public spaces. This suggests that places of worship should have a crime-reducing association in neighborhoods [6, 7, 9, 10], even after controlling for a range of factors known to be associated with aggregate crime outcomes.

Although there are many reasons to expect places of worship (POW) will reduce the mount of crime in neighborhoods [6, 7, 9], there is a dearth of empirical work to assess this proposition [7, 11]. A principal reason for this mismatch between theory and data is that geospatial information on churches is generally lacking [7, 11]. Because POW have tax-exempt status, they are not compelled to report to the Internal Revenue Service (IRS) or most municipalities, thereby it is a bedeviling challenge for researchers to accurately capture POW aggregated to microgeographic units. For example, even the National Center for Charitable Statistics (NCCS) does not have a directory or listing of all POW located across the United States (U.S.) [11]. Data issues aside, the few studies to have analyzed the effects of POW have provided weak evidence of their crime-reducing behavior [6, 7, 9, 12]. This raises the possibility that POW might signify an unintended consequence for neighborhood crime control.

The crime and place literature has established the crime tends to spatially concentrate at or near risky facilities, including bars, liquor stores, check-cashing stores, retail outlets, restaurants, schools, and more [13–17]. This is because such facilities generally provide an opportunity structure for crime, that is, there is a tendency for motivated offenders and suitable targets to converge in space and time alongside weak guardianship [18–20]. More specifically, these facilities have deleterious effects because they operate as *crime generators* [19, 21]. The latter is a foundational concept of environmental criminology and refers to the following:

“particular areas to which large number of people are attracted for reasons unrelated to any particular level of criminal motivation they might have or to any particular crime they might end up committing [19:7].”

Thus, the concept of a crime generator is predicated on high foot traffic (or what is referred to as a large ambient population) operating alongside emerging criminal opportunities. The implication is that potential offenders are not traveling to these areas with the specific intent to commit crime [18, 19]. Rather, potential offenders will recognize situations in which there is a weakly guarded target, while going about their routine activities, and seize these opportunities to commit crime [13, 19, 20]. Analogous to how schools, restaurants, and retail stores have been linked to more crime in place [13, 17, 22, 23], we propose that POW might unintentionally lead to neighborhood crime problems, mainly because of their ability to induce high foot traffic, which offers an abundance of targets, while at the same time undermining guardianship and social control capabilities [24, 25].

There are competing expectations for how places of worship might impact crime in place. On the one hand, the literature rooted in social capital suggests that POW are crime-reducing because they engender social ties, common values and goals, and a responsibility for the collective good [1, 3, 4]. Conversely, POW might unintentionally be associated with more crime because they produce high foot traffic and undermine guardianship and social control capabilities, consistent with the environmental criminology literature [18, 19]. Despite these competing expectations, there are few studies to empirically assess the POW-crime nexus in neighborhoods. Therefore, for the present study, we conduct a block group analysis of crime, places of worship, well-established criminogenic facilities, and sociodemographic characteristics in Washington, DC.

## POW and social capital

A voluminous body of literature highlights how local institutions facilitate networks of effective social action in terms of crime control [7, 11, 26–28]. Places of worship (POW) specifically have been argued to strengthen dimensions of social capital such as social ties, mutual cohesion and trust, and a willingness to intervene for the common good because of sponsored events and activities [2–4].

Consequently, POW can produce two types of social capital: 1) Bonding social capital builds intraneighborhood cohesion and social ties among adherents and residents, while 2) bridging social capital establishes interneighborhood cohesion and social ties among local institutions, municipal agencies, and other groups of people [4, 8]. Both bonding and bridging social capital are theorized to strengthen neighborhoods’ capacity for collective action against crime problems. So, when neighborhoods exhibit relatively higher levels of social capital (as a result of POW), there is a greater likelihood that residents and adherents will acknowledge crime problems, will achieve consensus on how to address these problems, and will solve the problems in a more collective fashion [2, 4, 8]. This leads to our first proposition:

**P1.** Places of worship will be associated with *lower* counts of both violent and property crime, controlling for a range of factors known to be associated with crime in neighborhoods.

The seminal work of Robert Putnam [4, 29] posits that successful outcomes (in terms of education, health, crime control, family structure, etc.) are more likely in communities with high social capital, with local institutions like churches being the catalyst for the latter. Putnam’s [4] analysis indeed reveals a negative effect of local institutions on crime in U.S. counties. Similarly, Lee (10) constructs an index of civic engagement, which includes an indicator of congregations, and finds that this index is negatively associated with crime in rural U.S. counties. In one of the more comprehensive examinations linking POW to crime in place, Beyerlein and Hipp (8) determine that three POW measures are largely associated with lower levels of murder, burglary, assault, and robbery in U.S. counties. Yet despite this evidence in support of proposition 1, there is also evidence to the contrary. For instance, one study failed to determine that POW are related to lower violent and property crime in New York (NY) block groups [7]. This study also failed to detect any conditional effects of their POW measure. Also, another study found that three church measures (i.e., church presence, total churches, and churches within 500 ft) had nonsignificant effects on informal social control in Louisville and Lexington (KY) block groups [9].

## POW and criminal opportunities

Environmental criminology has argued and shown that crime is spatially concentrated in neighborhoods with a high density of nonresidential activities [30–32]. More specifically, activity nodes refer to locations where people spend a significant amount of time conducting nonresidential routine activities (e.g., work, school, grocery, recreation, shopping, etc.), while pathways are features of the planned physical environment that connect activity nodes with one another (e.g., a road network, monorail or train system, bus lines, walking trails, etc.). Neighborhoods with a higher concentration of nodes and pathways have indeed been shown to have a disproportionate amount of crime [13, 16, 31] and this is because nodes and pathways yield overlapping activity and awareness spaces of large numbers of people—a recipe for crime problems [18–20].

Environmental criminologists have increasingly used the term *crime generator*, an extension of the activity node concept, to denote physical structural qualities of neighborhoods that breed crime as a result of foot traffic and anonymity specifically [13, 16, 33]. Stores, restaurants, and schools commonly operate as crime generators because of their ability to increase the volume of targets and undermine guardianship capabilities, that is, the ability to informally monitor and regulate public spaces [16, 17, 22–24, 34, 35]. Offenders do not travel to these locations with the specific intent to commit crime [19], rather, the key implication is that amid high foot traffic, a potential offender will notice a weakly guarded target (person or object) and seize the opportunity to commit crime.

Given that foot traffic is a defining characteristic of crime generators, we argue that POW might operate in the latter capacity. In addition to religious meetings and services that occur on a weekly basis, many POW have a mission statement that involves the provision of need-based social services to its members, as well as less fortunate people in the larger community. These services include shelter and housing, food/soup kitchens, therapy and counseling, and job procurement and training [7, 9, 12]. When POW are effective in improving the social circumstances of people, these people may be less inclined to resort to criminal behavior [7, 11], yet this remains an open question. On the other hand, high foot traffic because of the provision of need-based services will almost certainly induce criminal opportunities [6]. Analogous to how a shopping mall not only provides positive services and economic benefits but also provides criminal opportunities by increasing the presence of both potential offenders and targets [22], a place of worship has the ability to increase the number of potential offenders and targets in a neighborhood simply through the increased foot traffic (or ambient population) that results. We therefore evaluate a second proposition:

**P2.** Places of worship will be associated with *higher* counts of both violent and property crime, controlling for a range of factors known to be associated with crime in neighborhoods.

Two studies reveal that places of worship might unintentionally lead to crime problems in neighborhoods. Triplett, White (6) determine that neighborhood variation in street crimes and domestic assaults, respectively, are positively associated with the number of churches in Norfolk (VA) block groups. Desmond, Kikuchi (12) examine how different types of religious congregations are linked to crime in Indianapolis (IN) block groups. The authors determine that their congregation measures mainly have nonsignificant effects on violence whereas for property crime, most of these measures yield significant positive effects. Conversely, there are no instances in which the density of congregations shows a crime-reducing association (except for civically engaged organizations). However, both these studies do not control for the presence of well-established criminogenic facilities (e.g., bars, liquor stores, check-cashing stores, retail outlets), and therefore the observed criminogenic effects of POW might be attributed to the latter facilities.

In the sections that follow, we explain our data and analytic strategy used to test our propositions (i.e., P1 and P2). After reporting the findings, we provide a discussion of the implications for criminological theory, urban studies, public policy, and future research.

## Data and methods

### Study area

The study area is Washington, DC, the Capital of the United States. DC is an urban area with 670,050 persons according to the most recent estimate by the United States Census Bureau (https://www.census.gov/quickfacts/fact/table/DC,US/PST045221). It follows that DC is one of the most populous areas within the United States, specifically, ranking 23^rd^ among U.S. cities. DC also has an ethnically diverse population; 37.3% of residents self-identify as white, 45.8% as Black, 11.5% as Latino, and 4.5% as Asian. Although the median household income is significantly more than the national average ($90,842 versus $64,994), such disparity is explained by DC’s high cost of living, and therefore it is not surprising to observe that DC’s poverty rate exceeds that of the national average (16.5% versus 11.6%).

Washington, DC, offers a favorable setting for examining the effects of places of worship (POW) on crime in place for several reasons. First, DC has recently (and historically) exhibited rather high levels of both violent and property crime among the 100 most populous U.S. cities. In 2019, for example, DC ranked 24^th^ and 29^th^ in violent and property crime rates, respectively (https://www.fbi.gov/how-we-can-help-you/need-an-fbi-service-or-more-information/ucr/publications). Therefore, there is a need to determine the factors that affect the spatial distribution of crime problems in DC. This leads to a second reason: Washington, DC, has a large presence of facilities (e.g., alcohol stores, retail districts/centers, check-cashing stores, and metro stations) which have been theorized or shown to be associated with more crime in place [13, 16, 19]. The presence of various criminogenic facilities has been provided through DC’s open data portal (https://opendata.dc.gov/), and therefore this provides the necessary means to minimize the possibility of obtaining spurious effects of our key independent variable (i.e., places of worship) on crime. Furthermore, DC has a large presence of POW (N = 742) with the corresponding longitude (x) latitude (y) data made publicly available through the portal. Notably, previous researchers have documented the many challenges of collecting geospatial data on POW [7, 11, 26, 36]; most notably, there is not a census of POW in the U.S. because most of them are tax-exempt and therefore they are not accurately captured by data provided by the National Center for Charitable Statistics (NCCS). By using DC data on POW in combination with their large presence, it affords us the flexibility to test for a multitude of main and moderating effects.

### Units of analysis and sample

The U.S. Census Bureau provides data on various geographic/spatial units. We draw on block groups specifically to link places of worship to crime in place, primarily because block groups have been designed to be homogenous on a range of sociodemographic characteristics, including income and poverty, educational attainment, household structure, age, and length of residence [37, 38]. Thus, our selection of block groups as our units of analysis is consistent with previous empirical work that has examined “neighborhood effects” on a range of outcomes, such as ethnic and racial segregation [e.g. 39], social networks [e.g. 40], walkability and health [41], gentrification [e.g. 42], and crime [43], to name a few.

The present study involves secondary data analysis of block groups from publicly existing data, and therefore did not require institutional review board approval. For our analysis, we estimate crime models using a sample of 449 block groups (out of the 450 in DC); one block group has been dropped because it is missing necessary information from the U.S. Census American Community Survey (ACS). We cannot use constituent tract information as a substitute for missing block group information, because for this block group, the tract and block group boundaries are exactly the same. We suspect missing data for some variables is attributed to the fact that this area largely encompasses Georgetown University and its affiliated facilities.

### Dependent variables: Crime counts

We have collected crime data from DC’s open data portal. These are official crime data coded and reported by the District of Columbia Metropolitan Police Department (MPD). MPD has provided crucial information on each crime incident from 2021, including the longitude–latitude coordinates of the crime, the date in which the crime occurred, and the type of Part 1 crime committed according to the Uniform Crime Reporting (UCR) program in the United States. Accordingly, we aggregated these data to their constituent block group and computed the number of incidents for the following crime types: murders, robberies, aggravated assaults with a gun, burglaries, larcenies, and motor vehicle thefts. Furthermore, we created an index of *violent crimes* (combining murders, robberies, and assaults) along with an index of *property crimes* (combining burglaries, larcenies, and motor thefts). Notably, our main models utilize the latter two indices as outcome measures, whereas some of the ancillary models assess each of the crime types (separately) that comprise both indices.

### Independent variables: Places of worship, criminogenic facilities, and sociodemographic characteristics

DC’s open data portal provides information on places of worship in 2019, most notably, the longitude–latitude coordinates of each POW. We identified 742 POW in the dataset after eliminating 32 cases with coordinates outside of the study area or with duplicate coordinates. Although some prior studies have theorized that the effects of POW differ by the religion or denomination of POW [8, 9, 44], DC only classifies its POW by seven religions, of which 97% of them are determined to be Christian. Denomination information was not provided for places of worship. We also determined that the name of POW was not sufficient for accurately classifying POW into denominations. Thus, we have created an index of the number of all *places of worship*, aggregated to block groups.

One of the most enduring correlates of spatial crime patterns is the presence of facilities that provide an opportunity structure for offenders, targets, and weak guardianship to converge in space and time [18, 19]. Accordingly, we constructed several variables to capture such facilities. These facilities include the number of *onsite alcohol outlets* (i.e., bars, night clubs, and taverns), *offsite alcohol outlets* (i.e., liquor stores and convenience stores), *check-cashing stores*, and *retail districts/centers* (e.g., shopping malls and plazas). We also include a dichotomous variable for the presence of a *DC metro station* (1 = Yes and 0 = No).

It is also necessary to control for sociodemographic characteristics that have been linked to the spatial distribution of crime [25, 45–47]. Drawing on data from the U.S. Census Bureau we create measures of various sociodemographic characteristics. In particular, we utilize the American Community Survey (ACS) five-year estimates from 2015 to 2019, aggregated to block groups. To capture differences in economic hardship, we account for *poverty* (%) in block groups. We computed a Herfindahl index of five ethnic groups (white, Black, Latino, Asian, and other races) to account for the *ethnic heterogeneity* of block groups. The concentration of both *Black* (%) and *Latino* (%) residents are also included to account for populations that have been historically marginalized by the political economy of place [48]. Furthermore, we employ a variable of *homeowners* (%) as a proxy for residential stability and we control for two types of housing characteristics: *the number of housing units* (/100) and *occupied units* (%). Finally, we created a variable of the *population* (/100) along with a variable that specifically captures the age group with the highest rate of offending and victimization, that is, *persons aged 15 to 29* (%). Descriptive statistics for all measures are shown in Table 1.

**Table 1:** Summary statistics.

	Mean	SD
**Dependent Variables**		
Violent crimes	8.68	9.64
Property crimes	53.67	70.43
**Independent Variables**		
Places of Worship	1.65	2.01
% poverty	18.99	16.24
Ethnic heterogeneity	42.01	20.04
% Black	45.60	34.57
% Latino	10.58	10.31
% homeowners	46.86	26.77
# housing units /100	7.00	3.98
% occupied units	90.35	7.87
Population /100	15.39	7.67
% aged 15 to 29	23.08	13.57
Alcohol outlets (onsite)	1.12	3.52
Alcohol outlets (offsite)	.62	.95
Check-cashing stores	.24	.61
DC Metro Station	.08	.27
Retail districts/centers	.72	2.27
Lagged violent crimes	9.58	7.21
Lagged property crimes	61.76	48.41

Notes

SD = standard deviation

N(block groups) = 449.

### Analytic strategy

The dependent variables of crime counts are significantly skewed and overdispersed (i.e., the variance exceeds the mean). Thus, we analyze the spatial distribution of crime using negative binomial regression in Stata 17; a Poisson-based regression that effectively accounts for overdispersion via its alpha parameter [49, 50]. While the Poisson distribution can be appropriately used to model certain count variables, for the present study, we find that Stata’s likelihood-ratio test, which tests the null hypothesis that the dispersion parameter (alpha) is equal to zero, is significant for all our models (*p* < 0.05). Negative binomial regression is therefore needed to account for overdispersion [49, 50]. Yet at the same time, we acknowledge that ordinary least squares regression (OLS) is a viable alternative for modeling the spatial distribution of crime, especially given that a large majority of block groups do not have zero crime incidents, and therefore we have estimated ancillary models using OLS. To be clear, we are concerned with using the appropriate model(s) to analyze the spatial distribution of crime, however, it should be noted that negative binomial and OLS regression models are not representative of spatial regression. Therefore, we estimate ancillary spatial error models as a final robustness check (described in more detail below).

Geographic units such as block groups are not islands unto themselves [51], in fact, the conditions of spatially contiguous/adjacent units can very well shape what occurs in the focal unit—what is often referred to as a spillover effect. What this means for the current study is that crime in the focal block group is likely impacted by the amount of crime in nearby block groups [52–54]. To account for this spatial dependence, we constructed a spatially lagged measure for each crime outcome using GeoDa software with first-order queen contiguity. Such a measure captures the average number of crime incidents among contiguous block groups in relation to the focal block group. We include a spatially lagged measure of crime (as a predictor) in our full models.

A general expression of the (full) negative binomial regression models that we estimate is as follows:

$$y=B_{1}{POW}_{}+B_{2}{SD}_{}+B_{3}CF+B_{4}SLy+\alpha ,$$ (1)

where *y* is the number of crime incidents, POW is the number of places of worship, **SD** is a matrix of the sociodemographic characteristic measures, **CF** is a matrix of the criminogenic facility measures, SLy is the average number of crime incidents in block groups adjacent to the focal block group (a spatially lagged measure), and α is an intercept.

While one approach for modeling crime across geographic units is to specify the population count as an exposure term (thereby estimating the outcome as a crime rate), we have instead modeled crime counts by including the population count as a predictor, given growing concerns over population count being the denominator of a calculated crime rate [e.g., see 18, 55–58]. As anticipated, we detected minimal evidence of spatial autocorrelation in our full models as a result of including the spatially lagged measure of crime. Although the Moran’s I value was statistically significant in all instances, the maximum value was .08 (which is rather weak given that positive spatial autocorrelation ranges from 0 to 1). Furthermore, we assessed and found no evidence of multicollinearity issues based on variance inflation factors (VIF). The maximum VIF was 4.68, which does not exceed the commonly used cutoff of 10 [59, 60].

In the results section, we present two models for both the violent and property crime outcomes (Table 2). We first estimate a *baseline* model that features our places of worship measure along with the sociodemographic characteristic measures, consistent with the modeling approach undertaken by certain prior studies [for example, see 6, 8, 12]. We then estimate a *full model* that additionally includes the measures of well-established criminogenic facilities and the spatially lagged measure of crime in order to determine the extent to which places of worship maintains a significant effect on crime (if at all). Crime and place researchers have called for analyses to integrate measures associated with social disorganization and routine activities theories simultaneously [for example see, 33, 61]; therefore, our full model is consistent with this call.

**Table 2:** POW effects on neighborhood crime.

	M1:Violent	M2:Violent	M3:Property	M4:Property
	b/se	b/se	b/se	b/se
Places of Worship	.121**	.059**	.136**	.066**
	(.020)	(.017)	(.017)	(.015)
% poverty	-.003	-.001	-.005	-.004
	(.004)	(.003)	(.003)	(.003)
Ethnic heterogeneity	.005+	.002	.005*	.000
	(.003)	(.002)	(.002)	(.002)
% Black	.018**	.014**	-.000	.001
	(.002)	(.002)	(.001)	(.001)
% Latino	.001	.005	-.003	.003
	(.005)	(.004)	(.004)	(.004)
% homeowners	-.008**	-.004*	-.003+	-.001
	(.002)	(.002)	(.002)	(.002)
# housing units /100	.059**	.021	.085**	.034*
	(.021)	(.019)	(.019)	(.017)
% occupied units	-.007	-.006	-.007	-.007
	(.005)	(.005)	(.005)	(.004)
Population /100	.012	.015+	.006	.014+
	(.010)	(.009)	(.009)	(.008)
% aged 15 to 29	.007	.001	.008*	-.001
	(.004)	(.004)	(.003)	(.003)
Alcohol outlets (onsite)		.030**		.033**
		(.011)		(.011)
Alcohol outlets (offsite)		.156**		.132**
		(.047)		(.043)
Check-cashing stores		.106+		.117*
		(.058)		(.055)
DC Metro Station		.242*		.281**
		(.123)		(.109)
Retail districts/centers		.005		.030
		(.019)		(.020)
Lagged violent crimes		.045**		
		(.006)		
Lagged property crimes				.004**
				(.001)
Intercept	.990+	.665	3.470**	3.329**
	(.596)	(.512)	(.525)	(.447)

Notes: N(block groups) = 449.

b = unstandardized coefficient, (SE) = standard error.

+ p<0.10

* p<0.05

** p<0.01

In addition to discussing the observed effects in terms of their direction and statistical significance, we highlight the magnitude of these effects in relation to one another. We draw on an approach that determines the percent change in the expected crime count *for a one standard deviation* increase in the variable of interest using the following formula: (exp(β× SD)– 1) *100. This is a preferred approach because some of our independent variables drastically differ in terms of their scales [49: 492–493, 514–516.], most notably, the POW and facility measures are counts whereas the sociodemographic characteristic measures are percentages. Similar to previous crime and place studies [62–65] we utilize this approach to effectively compare the effect sizes of variables with substantively different scales.

On the other hand, we recognize that another common approach is to assess the magnitude of the effects using incident rate ratios (IRR). Specifically, an IRR denotes the percent increase or decrease *for every one-unit increase* in a predictor by multiplying the difference between the IRR and one by 100 where positive values yield a percent increase and negative values yield a percent decrease [49]. In Table 3, we compute the effect sizes using both approaches, although we base our inferences on the first approach because for the second approach, a one-unit increase may represent a very large increase for one predictor (e.g., DC Metro Station) and a very small increase for another predictor (e.g., population).

**Table 3:** The substantive effects of predictors from full models.

	Violent Crimes		Property Crimes	
Predictors	Model 2 SD Effect	Model 2 IRR	Model 4 SD Effect	Model 4 IRR
Places of Worship	12.6	1.06	14.2	1.07
% poverty				
Ethnic heterogeneity				
% Black	59.8	1.01		
% Latino				
% homeowners	-10.3	0.995		
# housing units /100			14.3	1.03
% occupied units				
Population /100	12.4	1.02	11.5	1.01
% aged 15 to 29				
Alcohol outlets (onsite)	11.1	1.03	12.4	1.03
Alcohol outlets (offsite)	16	1.17	13.4	1.14
Check-cashing stores	6.7	1.11	7.4	1.12
DC Metro Station	6.7	1.27	7.8	1.32
Retail districts/centers				
Lagged violent crimes	38.6	1.05		
Lagged property crimes			24.3	1.04

*Notes*: SD Effect size refers to the percent change (%) in the expected crime count using the formula [exp(β X SD)– 1]100, on an all other things equal basis. IRR refers to the incident rate ratio effect size.

Empty cells denote non-significant effects.

## Results

### Violent crimes

Model 1, a baseline model, shows that places of worship (POW) is significantly and positively associated with violent crime counts, controlling for sociodemographic characteristics that are commonly accounted for by crime and place researchers [45]. A 1 standard deviation (SD) increase in places of worship implies a 27.6% *increase* in the expected number of violent crimes based on the following formula: (exp(β× SD)– 1) *100.

We also find that ethnic heterogeneity is positively associated with violent crime, albeit at the marginally significant threshold (p < .10), while the percentage of Black residents also shows a positive association with the outcome. The percentage of homeowners is significantly and negatively associated with violent crime, whereas the number of housing units exhibits the opposite relationship. This baseline model suggests that POW might have an unintended consequence, consistent with proposition 2.

To minimize the possibility of obtaining spurious effects of POW, it is necessary to account for physical structural qualities of neighborhoods that have been linked to crime. In model 2, we therefore include measures of various facilities as well as the spatially lagged measure of violent crime counts. Although ethnic heterogeneity is found to be no longer related to violent crime, the positive relationship between the percentage of Black residents and violent crime persists. Moreover, the negative relationship between homeownership and violent crime remains as well. On the other hand, the positive effect of housing units becomes nonsignificant, whereas population is now related to more violent crime (albeit at the marginally significant threshold). Most of the facility measures indicate significant relationships with violent crime in the hypothesized direction, that is, violent crime in block groups is positively linked to the presence of alcohol outlets (both onsite and offsite establishments), check-cashing stores, and the presence of DC metro stations, respectively. We also determine a positive relationship between the spatially lagged measure and the outcome. What this means is that violent crime in nearby block groups is related to higher numbers of violent crime in the focal block group.

Similar to model 1, we observe that POW maintains its significant and criminogenic effect in block groups. Yet, the size of the coefficient from model 1 to model 2 has decreased by more than half (from .121 to .059). Thus, the effect of POW on violent crime is much weaker as well: There is a 12.6% increase in the number of violent crimes for a 1 SD increase in POW. Although the inclusion of the facility and spatial lag measures naturally reduces the POW effect in terms of magnitude, the latter has one of the strongest effects among the variables in the full model (see Table 3). The magnitude of the effect of POW, specifically, rivals or even outpaces those of on-premise alcohol outlets, off-premise alcohol outlets, check-cashing stores, and the presence of a DC metro station. All of this is to say is that the full model of violent crime reinforces the notion that POW appear to operate as crime generators (not social capital organizations).

### Property crimes

Model 3, a baseline model, reveals that places of worship is significantly and positively related to higher numbers of property crime in block groups, controlling for sociodemographic characteristics that have been linked to crime in place. The magnitude of this effect is quite strong: A 1 SD increase in places of worship implies a 31.4% *increase* in the expected number of property crimes, consistent with proposition 2. While most of the sociodemographic characteristic measures have nonsignificant effects, we do find some instances in which they do indeed affect the spatial distribution of property crimes. For instance, we detect positive effects for ethnic heterogeneity, number of housing units, and the percent aged 15 to 29. On the other hand, percent homeowners exhibits a (marginally significant) negative effect on property crime.

In model 4, we additionally include the facility measures along with the spatially lagged measure of property crime. This provides a conservative test of the effect of POW on property crime in block groups. We find that the effects of ethnic heterogeneity, the percent homeowners, and the percent aged 15 to 29 are no longer significant in the full model. Conversely, the number of housing units maintains its positive effect, while the population measure now shows a marginally positive association with the outcome. Consistent with environmental criminology [18, 19], each of the facility measures have a positive effect on property crime (except for the presence of retail districts/centers). We also find evidence of property crime being spatially clustered, as the spatially lagged measure indicates a positive effect.

The coefficient estimate for POW has decreased by more than half from the baseline to the full model (from .136 to .066), which is similar to what we observed for violent crime. Nonetheless, the criminogenic effect of POW remains strong; there is a 14.2% increase in the number of property crimes for a 1 SD increase in POW. Moreover, the magnitude of this effect is stronger than all the other predictors in the model (Table 3) with the exception of housing units and the spatially lagged measure of property crime. Because POW have strong positive effects on both violent and property crimes, this suggests that POW may need to be reconceptualized as a crime generator rather than a source of social capital that is leveraged to reduce crime.

## Ancillary results

An alternative analytic strategy is to model the violent and property outcomes using ordinary least squares regression (OLS) instead of negative binomial regression. Thus, we have estimated OLS models of violent and property crimes using the same model specifications as those implemented for negative binomial regression (i.e., Table 2, M1-M4). Table 4 shows the results of the OLS models, and it is apparent that the pattern of results is very similar to those produced by negative binomial regression. Although the criminogenic effect of POW weakens in magnitude between the baseline and full model for both violent and property crimes, it nonetheless remains statistically significant and crime-producing. Also, the measures capturing sociodemographic characteristics and well-established criminogenic facilities yield effects that are virtually the same as those produced by negative binomial regression (in terms of both direction and statistical significance).

**Table 4:** Ordinary least squares regression: POW effects on neighborhood crime.

	M1:Violent	M2:Violent	M3:Property	M4:Property
	b/se	b/se	b/se	b/se
Places of Worship	.983**	.409*	9.809**	3.040**
	(.178)	(.167)	(1.370)	(1.036)
% poverty	.017	.025	-.383	-.173
	(.034)	(.030)	(.259)	(.185)
Ethnic heterogeneity	-.016	-.030	.437*	.112
	(.026)	(.023)	(.199)	(.147)
% Black	.099**	.080**	.040	.135
	(.015)	(.015)	(.118)	(.084)
% Latino	-.040	-.015	-.491	-.189
	(.045)	(.041)	(.349)	(.254)
% homeowners	-.043*	-.014	-.133	.067
	(.019)	(.018)	(.150)	(.108)
# housing units /100	.556**	.158	7.467**	2.359*
	(.179)	(.166)	(1.380)	(1.030)
% occupied units	-.031	-.017	-.482	-.264
	(.049)	(.043)	(.376)	(.268)
Population /100	.247**	.311**	.498	1.521**
	(.089)	(.080)	(.683)	(.493)
% aged 15 to 29	.020	-.018	.302	-.209
	(.035)	(.032)	(.272)	(.196)
Alcohol outlets (onsite)		.330**		7.604**
		(.124)		(.766)
Alcohol outlets (offsite)		1.315**		8.081**
		(.478)		(2.950)
Check-cashing stores		1.294*		2.920
		(.603)		(3.716)
DC Metro Station		1.878		13.802+
		(1.258)		(7.757)
Retail districts/centers		.429*		6.494**
		(.191)		(1.179)
Lagged violent crimes		.244**		
		(.059)		
Lagged property crimes				.136**
				(.045)
Intercept	-.056	-2.201	12.611	.226
	(5.352)	(4.784)	(41.273)	(29.425)

Notes: N(block groups) = 449.

b = unstandardized coefficient, (SE) = standard error.

+ p<0.10

* p<0.05

** p<0.01

Another line of inquiry that is necessary to investigate is whether POW affect certain crime types differently. We therefore estimated separate models for each crime type that comprises the violent and property crime indices. From Table 5, we determine that POW is significantly and positively associated with the number of robberies (14.5% more for a 1 SD increase), burglaries (12.2% more for a 1 SD increase), larcenies (13.8% more for a 1 SD increase), and motor vehicle thefts (15.3% more for a 1 SD increase), while demonstrating a marginally significant association with murders in the same direction (18.5% more for a 1 SD increase). So, aggravated assault is the lone instance by which POW fails to have a statistically significant relationship with a form of crime. Finally, we did test for moderating effects between certain independent variables (e.g., POW X poverty and POW X percent Black), but in all instances these interaction terms were found to be nonsignificant.

**Table 5:** Negative binomial regression: POW effects on neighborhood crime.

	Murder	Robbery	Assault	Burglary	Larceny	Motor
	b/se	b/se	b/se	b/se	b/se	b/se
Places of Worship	.085+	.067**	.029	.057*	.064**	.071**
	(.044)	(.019)	(.021)	(.025)	(.016)	(.018)
% poverty	.008	-.006+	.006	.003	-.004	-.009*
	(.008)	(.004)	(.004)	(.005)	(.003)	(.003)
Ethnic heterogeneity	.007	.001	.005	-.001	.001	-.002
	(.008)	(.003)	(.003)	(.004)	(.002)	(.003)
% Black	.031**	.011**	.019**	.002	-.000	.009**
	(.006)	(.002)	(.002)	(.002)	(.001)	(.002)
% Latino	.008	.003	.009+	.003	.002	.004
	(.012)	(.005)	(.005)	(.006)	(.004)	(.005)
% homeowners	-.008	-.002	-.005+	-.001	-.000	-.003
	(.005)	(.002)	(.002)	(.003)	(.002)	(.002)
# housing units /100	.013	.017	.040+	.032	.032+	.033+
	(.051)	(.021)	(.022)	(.027)	(.018)	(.020)
% occupied units	-.003	-.006	-.003	-.002	-.007+	-.003
	(.012)	(.005)	(.006)	(.007)	(.004)	(.005)
Population /100	.006	.017+	.010	.015	.016+	.010
	(.022)	(.010)	(.011)	(.013)	(.008)	(.009)
% aged 15 to 29	-.002	.003	.000	.003	-.002	.004
	(.010)	(.004)	(.005)	(.005)	(.003)	(.004)
Alcohol outlets (onsite)	.034	.017	.047**	.017	.031**	.048**
	(.029)	(.013)	(.013)	(.016)	(.012)	(.012)
Alcohol outlets (offsite)	.172	.154**	.141*	.225**	.133**	.086+
	(.126)	(.054)	(.058)	(.068)	(.044)	(.051)
Check-cashing stores	.057	.124+	.125+	.073	.116*	.116+
	(.145)	(.065)	(.070)	(.087)	(.058)	(.062)
DC Metro Station	-.186	.273+	.164	.014	.317**	.035
	(.366)	(.141)	(.153)	(.180)	(.113)	(.130)
Retail districts/centers	-.002	.011	.003	.002	.030	.040+
	(.047)	(.021)	(.021)	(.025)	(.021)	(.021)
Lagged murders	.068					
	(.177)					
Lagged robberies		.094**				
		(.012)				
Lagged assaults			.080**			
			(.015)			
Lagged burglaries				.102**		
				(.022)		
Lagged larcenies					.006**	
					(.001)	
Lagged motor thefts						.026**
						(.005)
Intercept	-3.126*	.250	-.973	-.285	3.193**	.980+
	(1.440)	(.595)	(.646)	(.751)	(.465)	(.550)

Notes: N(block groups) = 449.

b = unstandardized coefficient, (SE) = standard error.

+ p<0.10

* p<0.05

** p<0.01

A final consideration is to determine whether the pattern of results remains unchanged when estimating spatial regression models, given that negative binomial regression and OLS regression do not explicitly account for spatial autocorrelation. We therefore estimate a spatial error model for both the violent and property outcomes, thereby mimicking the full model specifications illustrated in Table 2. Specifically, we draw on GeoDa software to conduct maximum likelihood estimation of spatial error models that include a spatial autoregressive error term. Table 6 shows the results of the two spatial error models, and it is apparent that the pattern of results is very similar to those produced by negative binomial regression and OLS regression. Not only is POW significantly and positively related to violent and property crimes, but also the Moran’s I of the spatial error residuals are very close to zero. This means that including the spatially autoregressive error term has in effect removed all the spatial autocorrelation from these models.

**Table 6:** Spatial error models, maximum likelihood estimation: POW effects on neighborhood crime.

	M1:Violent	M2:Property
	b/se	b/se
Places of Worship	.492**	3.223**
	(.168)	(1.026)
% poverty	.033	-.140
	(.030)	(.182)
Ethnic heterogeneity	-.030	.193
	(.025)	(.147)
% Black	.106**	.140
	(.016)	(.089)
% Latino	-.010	-.298
	(.042)	(.253)
% homeowners	-.015	.041
	(.017)	(.105)
# housing units /100	.284+	2.842*
	(.155)	(.941)
% occupied units	.006	-.189
	(.036)	(.224)
Population /100	.244**	1.296**
	(.074)	(.447)
% aged 15 to 29	-.002	-.160
	(.032)	(.194)
Alcohol outlets (onsite)	.329**	7.643**
	(.123)	(.753)
Alcohol outlets (offsite)	1.368**	8.194**
	(.467)	(2.891)
Check-cashing stores	1.218*	3.061
	(.587)	(3.642)
DC Metro Station	2.062+	13.565+
	(1.243)	(7.664)
Retail districts/centers	.493**	6.670**
	(.189)	(1.163)
Intercept	-3.672	-.227
	(4.055)	(24.865)
Spatial autoregressive coefficient (Lambda)	.242**	.122
	(.072)	(.077)
Moran’s I of residuals	-.010	-.002

Notes: N(block groups) = 449.

b = unstandardized coefficient, (SE) = standard error.

+ p<0.10

* p<0.05

** p<0.01

## Discussion

Physical structural qualities of neighborhoods have been argued to have consequences for crime [2, 18, 20], yet there is a dearth of research investigating how places of worship shape spatial crime patterns [6, 9]. What’s more, there are competing theoretical arguments for how POW would impact crime in neighborhoods, with social capital and environmental criminology perspectives arguing that POW have negative and positive associations, respectively. Accordingly, we examined the spatial distribution of violent and property crime in Washington (DC) block groups as a function of places of worship, well-established criminogenic facilities, and sociodemographic characteristics. We highlight three key findings.

The first key finding was that we consistently found POW to be associated with more violent and property crime, consistent with the results of two previous studies [6, 12]. This coupled with the fact we failed to detect a single instance of POW having a significant and crime-reducing effect suggests that POW do, in fact, operate as crime generators in neighborhoods. That is, the presence of outsiders lessens familiarity and makes it more challenging for insiders (i.e., members and residents) to identify potential offenders and detect suspicious, crime-related activity [18, 19, 24]. For insiders, there may be ambiguity concerning whom they should direct territorial behavior at, but also, the situational contexts by which it is acceptable to do so [24, 66, 67]. Recent studies have drawn on social media, cell phone, and transportation data to measure various properties of the ambient population and have indeed determined that the size of the ambient population is associated with more crime in place [56, 58, 68–70]. Thus, we propose that a significant increase in the volume of potential offenders and targets is enough to disrupt (or completely negate) a process whereby social capital is formed in POW and later used to instrumentally prevent and solve crime problems.

The second key finding was that places of worship exerted strong criminogenic effects, even after controlling for well-established criminogenic facilities and sociodemographic characteristics. We found that the effects of POW were stronger in magnitude than most of the other predictors in the violent and property crime models (Table 3), including alcohol outlets with onsite consumption, check-cashing stores, and the presence of a DC Metro station. Moreover, ethnic heterogeneity and poverty, two predictors often equated with social disorganization [45, 46], were not significantly related to more crime. Because POW are important local institutions insofar as they promote education, steady employment, marriage, drug and substance avoidance, and friendships among members, our findings should not be interpreted as an indictment on religion or POW. Rather, it highlights POW as an (unexpected) ecological risk factor for neighborhood crime, similar to how shopping malls, central business districts, restaurants, and retail stores have been deemed to operate as crime generators [13, 16, 22, 35].

Our results have implications for both researchers and policymakers. When modeling crime across geographic units, crime and place researchers importantly control for factors that induce criminal opportunities (i.e., liquor stores, bars, check-cashing stores, and transit stops). We suggest additionally controlling for POW to minimize the possibility of obtaining spurious effects with regards to the independent variables of interest. Relatedly, for crime policy, we encourage researchers and city officials to account for the presence of POW in determining the risk of crime across areas within a city. This could be accomplished via two data driven approaches: 1) combine regression analysis and the mapping of predicted outcome variables [e.g., see 71], and 2) implement risk terrain modeling [e.g., see 72]. Many policing strategies and intervention efforts are predicated on identifying areas with a disproportionate amount of crime; therefore, the incorporation of POW may provide more accurate profiles on which areas would benefit the most from increased (fair and consistent) policing, or municipal resources, services, and partnerships.

### Limitations and directions for future research

Although the current study provides crucial insight into places of worship and crime in neighborhoods, we acknowledge certain limitations and directions for future research. First, because of data limitations we were unable to test the theorized mechanisms that may link POW to violent and property crime in neighborhoods (although this is true of nearly all prior studies on the topic). Thus, we encourage future research to collect neighborhood level data on social capital, civic engagement, foot traffic (or the ambient population), and anonymity in order to test whether these factors do, in fact, mediate the effects of POW on crime. Second, our key independent variable captures the presence of places of worship, and therefore it does not capture the differential capacity of POW to impact crime. A natural extension is for future studies to assess variation in neighborhood crime as a function of more fine-grained characteristics of POW, such as the number of adherents/members, employees, income/donations, and years of operation. A few studies have explored this line of inquiry [6, 11, 27], yet it remains to be seen whether measures that account for the differential capacity of POW provide additional knowledge beyond what can be gained from the standard measurement approach (i.e., the number of POW in a neighborhood). Also, it is an open question the extent to which DC’s data on POW is exhaustive and accurate, though we have no reason to suspect that these data are any less valid than other POW sources (e.g., Yelp, the phonebook, Google, etc.). And, there is no reason to think that any missing data is not random or systematic. Third, the analysis is cross-sectional; and therefore, it is unable to test how changes in the number of POW influence changes in the number of crimes. While this does not repudiate the findings of the present study, as understanding the spatial distribution of crime at a single timepoint offers an important baseline, future research may want to perform a longitudinal analysis using a fixed-effects approach. Finally, the analysis and findings pertain to neighborhoods of a single city. It is therefore possible that the observed effects might operate differently across U.S. cities. Accordingly, future work needs to examine potential relationships between places of worship and crime across a diversity of ecological settings, including cities beyond the United States.
